# Breast Milk Enema and Meconium Evacuation Among Preterm Infants: A Randomized Clinical Trial

**DOI:** 10.1001/jamanetworkopen.2024.7145

**Published:** 2024-04-22

**Authors:** Liqiang Zheng, Li Gai, Yani Wu, Chaonan Kong, Fangli Sun, Jinyue Gao, Wei Yuan, Min Liu, Hong Jiang, Nan Tuo, Fan Yang

**Affiliations:** 1 Clinical Research Centre, The International Peace Maternity and Child Health Hospital, Shanghai Jiao Tong University School of Medicine, Shanghai, China; 2 MOE-Shanghai Key Laboratory of Children’s Environmental Health, Xin Hua Hospital Affiliated to Shanghai Jiao Tong University School of Medicine, Shanghai, China; 3 School of Public Health, Shanghai Jiao Tong University School of Medicine, Shanghai, China; 4 Department of Pediatrics, Shengjing Hospital of China Medical University, Shenyang, Liaoning Province, China; 5 Department of Nursing, Shengjing Hospital of China Medical University, Shenyang, Liaoning Province, China; 6 School of Public Health, China Medical University, Shenyang, China

## Abstract

**Importance:**

Delayed meconium evacuation and delayed achievement of full enteral feeding among premature infants are associated with poor short- and long-term outcomes. Identifying a more effective and safer enema for meconium evacuation is imperative for improving neonatal care.

**Objective:**

To examine whether breast milk enemas can shorten the time to complete meconium evacuation and achievement of full enteral feeding for preterm infants.

**Design, Setting, and Participants:**

This randomized, open-label, parallel-group, single-center clinical trial was conducted from September 1, 2019, to September 30, 2022, among 286 preterm infants with a gestational age of 23 to 30 weeks in the neonatal ward of the Shengjing Hospital of China Medical University in Shenyang.

**Interventions:**

Preterm infants were randomly assigned to receive either breast milk enemas or normal saline enemas 48 hours after birth.

**Main Outcome and Measures:**

The primary outcomes were time to complete meconium evacuation and time to achieve full enteral feeding. Secondary outcomes were duration of hospitalization, weight at discharge, and duration of total parenteral nutrition. Intention-to-treat and per-protocol analyses were conducted.

**Results:**

In total, 286 preterm infants (mean [SD] gestational age, 198.8 [7.9] days; 166 boys [58.0%]) were eligible and included in this study. A total of 145 infants were randomized to the normal saline group, and 141 were randomized to the breast milk group. The time to achieve complete meconium evacuation was significantly shorter in the breast milk group than in the normal saline group (–2.2 days; 95% CI, −3.2 to −1.2 days). The time to achieve full enteral feeding was also significantly shorter in the breast milk group than in the normal saline group (−4.6 days; 95% CI, −8.0 to −1.2 days). The duration of total parenteral nutrition was significantly shorter in the breast milk group than in the normal saline group (−4.6 days; 95% CI, −8.6 to −1.0 days). There were no clinically notable differences in any other secondary or safety outcomes between the 2 groups.

**Conclusions and Relevance:**

In this randomized clinical trial testing the effects of breast milk enema on meconium evacuation, breast milk reduced the time to achieve complete meconium evacuation and achieve full enteral feeding for preterm infants with a gestational age of 23 to 30 weeks. Subgroup analyses highlight the need for tailored interventions based on gestational age considerations.

**Trial Registration:**

isrctn.org Identifier: ISRCTN17847514

## Key Points

**Question:**

Does breast milk enema facilitate meconium evacuation compared with normal saline for preterm infants?

**Findings:**

In this randomized clinical trial of 286 preterm infants with a gestational age of 23 to 30 weeks, the administration of breast milk enema resulted in a reduction in the time to achieve complete meconium evacuation and the time to achieve full enteral feeding compared with normal saline. No safety issues emerged.

**Meaning:**

The results of this trial demonstrated that breast milk, as a new enema agent, exhibited both effectiveness and safety compared with normal saline.

## Teaser

This randomized clinical trial examines whether breast milk enemas can shorten the time to complete meconium evacuation and achievement of full enteral feeding for infants born preterm in China.

## Introduction

For premature infants, establishing enteral feeding is a challenge in the neonatal intensive care unit,^1,2,3^ with delayed full enteral feeding associated with poor short- and long-term prognoses.^4,5,6^ This delay was related to the prolonged meconium excretion time caused by meconium retention^7,8^ due to immature intestinal motility mechanisms and neurotransmitter systems. Meconium leads to intestinal function blockage and gastrointestinal dysfunction.^9,10,11,12,13,14^ Furthermore, delayed meconium excretion prolongs the total parenteral nutrition (TPN) and hospital stay and increases the risk of infection. Studies have indicated that expediting meconium elimination can enhance feeding tolerance, facilitating a quicker transition to enteral nutrition and its associated benefits.^12^

Previous studies have shown many methods for promoting meconium evacuation, including abdominal massage, prophylactic rectal stimulation, oral contrast, and enema,^12,15,16,17,18^ of which enemas are the most common in clinical practice. Commonly used enemas include saline and glycerin solutions or suppositories. However, meta-analyses have shown that prophylactic glycerin suppositories, small-volume glycerin or normal saline enemas, or oral osmotic contrast agents to evacuate meconium did not reduce the time to reach full feeding in preterm infants.^19,20^ Furthermore, a case report described an infantile case of generalized urticaria caused by a glycerin enema solution,^21^ and a randomized clinical trial (RCT) found that a contrast agent may increase the risk of necrotizing enterocolitis (NEC).^18^ These findings led us to doubt the safety of glycerin and contrast agents for the enemas of premature infants. Therefore, new enemas must be identified to promote meconium evacuation among premature infants.

Human milk is an ideal source of nutrition and contains key immune-developing bioactive ingredients.^22^ The osmotic pressure of breast milk is appropriate^23^ because it is a benign stimulus to the digestive tract of premature infants. To our knowledge, saline enemas are safer than enemas with glycerin and contrast agents, and no adverse reactions have been reported. Therefore, this study used normal saline enemas as the control to explore whether breast milk enemas can shorten the time to complete meconium evacuation and reach full enteral feeding among premature infants. To our knowledge, this is the first study to use breast milk as an enema for premature infants. This RCT tested the hypothesis that, compared with normal saline, breast milk enema would shorten the time to complete meconium evacuation and reach full enteral feeding among premature infants with a gestational age of less than 30 weeks.

## Methods

### Study Design and Participants

This study was a randomized, open-label, parallel-group, single-center clinical trial conducted in the neonatal ward of the Shengjing Hospital of China Medical University in Shenyang from September 1, 2019, to September 30, 2022 (trial protocol and statistical analysis plan in Supplement 1). Neonates meeting the following criteria were enrolled: gestational age of less than 30 weeks and informed consent form signed by the guardian. Neonates were excluded if they had congenital malformations, congenital gastrointestinal anomalies, anorectal deformities, diarrhea, intussusception, NEC, patent ductus arteriosus, sepsis, neutropenia, and coagulopathy. The trial protocol^24^ was approved by the medical ethics committee of Shengjing Hospital, China Medical University. All relevant ethical regulations were followed. This study followed the Consolidated Standards of Reporting Trials (CONSORT) reporting guideline and statement.^25^

### Randomization and Blinding

Randomization was stratified by gestational age (23 weeks to <28 weeks, 28 weeks to <29 weeks, and 29 weeks to <30 weeks). Computer-generated randomization was conducted by an uninvolved statistician. The participants were then assigned to the intervention and control groups in a 1:1 ratio using a randomized block design. Participants were randomly assigned using opaque, sealed envelopes. Health care professionals administering the enemas were not blinded because of the nature of the intervention. However, the outcome assessors and statisticians were blinded to the group assignments.

### Procedures

Premature infants in the control and intervention groups received normal saline and breast milk enemas, respectively. Nurses were trained to ensure the standardization and safety management of enemas. The specific procedure was as follows: (1) Forty-eight hours after birth, preterm infants in the intervention and control groups received enemas twice a day at 9 am and 9 pm. (2) Material preparation included silicone tube (5 cm, model 3.33 mm [F10]), 5-mL syringe, thermostat (temperature setting 37 °C), sesame oil, and sterile gloves. (3) The required volume of the enema (5 mL/kg) was calculated and preheated to 37 °C in a thermostatically controlled water bath. Preheated enemas were extracted using a syringe. One end of the silicone tube was connected to a syringe, and the other end was lubricated with sesame oil and then slowly and gently inserted through the anus. The enema was slowly injected into the rectum for 3 minutes, and then the silicone tube was slowly removed.

Criteria for termination of the intervention included complete meconium evacuation, discontinuation of the intervention for infants experiencing adverse events (AEs) deemed unsuitable to proceed, or withdrawal of the guardian’s consent. The feeding and parenteral nutrition procedures in the control and intervention groups are detailed in the protocol.^24^ Other than the different enemas, there were no differences in treatment strategies or nursing methods between the control and intervention groups. The Trial Steering Committee dynamically monitored the rate of loss to follow-up.

### Outcomes

Two primary outcomes were assessed. The first was the time to achieve complete meconium evacuation, defined as the time until the occurrence of yellow stool for 1 day, encompassing both the complete evacuation of meconium and the transitional stool phase. The second primary outcome was time to complete enteral feeding, defined as the number of days from birth to full enteral feeding. More specifically, regarding full enteral feeding, criteria included the feeding volume of 180 mL/kg/d and weight gain exceeding 20 to 25 g/d within a 24-hour time frame. We accurately recorded the time to the hour and subsequently converted it into days by dividing it by 24 hours. The secondary outcomes were duration of hospitalization (in days), weight at the time of discharge, and duration of TPN. Safety outcomes included the incidence of AEs, including retinopathy of prematurity (any stage), intraventricular hemorrhage (grade 2), NEC, bronchopulmonary dysplasia, late-onset sepsis, and colorectal and anal injuries, as well as death.

### Data Collection, Management, and Quality Control

Trained researchers collected data using standardized questionnaires and measurements to ensure accuracy. At baseline, the following information regarding the premature infants and their mothers was collected: gestational age, birth weight, demographic information, history of disease, and medications used during pregnancy. The following major aspects of outcome information were collected during the follow-up: meconium evacuation, feeding, laboratory test results, and AEs. The data were securely managed throughout the trial and reviewed by quality control experts. Reasons were provided when data was modified. EpiData, version 3.1 (EpiData Association) statistical software was used to build the database.

### Statistical Analysis

Based on our pilot study, the breast milk enema group exhibited complete meconium evacuation 1.4 days, 1.2 days, and 1.2 days earlier than the saline enema group for the infants aged 23 weeks to less than 28 weeks, 28 weeks to less than 29 weeks, and 29 weeks to less than 30 weeks, respectively. With the use of a 2-sided significance level of .05, power of 0.8, and SD of 2, with a sampling ratio of 1 and factoring in a 20% dropout rate, the planned total sample sizes for infants aged 23 weeks to less than 28 weeks, 28 weeks to less than 29 weeks, and 29 weeks to less than 30 weeks were 78, 108, and 108 preterm infants, respectively (PASS, version 15.0; NCSS Co). Intention-to-treat and per-protocol analyses were conducted. We present the per-protocol results in eTable 4, eTable 5, and eTable 6 in Supplement 2. Descriptive statistics for all baseline characteristics are reported for each group. Categorical variables are presented as counts and percentages, while continuous variables are presented as mean (SD) or median (IQR) values. The Mann-Whitney test was used to compare the 2 groups according to the intention-to-treat and per-protocol principles for the primary and secondary outcomes. Outcomes are presented as median (IQR) values. For outcome differences between the treatment and control arms, the median difference and 95% CI were assessed using the Mann-Whitney test and the Hodges-Lehmann method. The primary outcomes were analyzed using Kaplan-Meier curves and log-rank tests. Safety was assessed by the number of AEs. Given that some infants discontinued due to AEs, sensitivity analyses were performed using worst outcome imputation and last observation time in the Mann-Whitney test.

Statistical significance was set at a 2-tailed *P* < .05. Due to the 2 primary end points, a Bonferroni correction was applied to our analyses of the primary outcomes. All statistical analyses were performed using IBM SPSS Statistics for Windows, version 22.0 (IBM Corp) and R Studio, version 4.1.2 (R Project for Statistical Computing).

## Results

**Figure 1.**
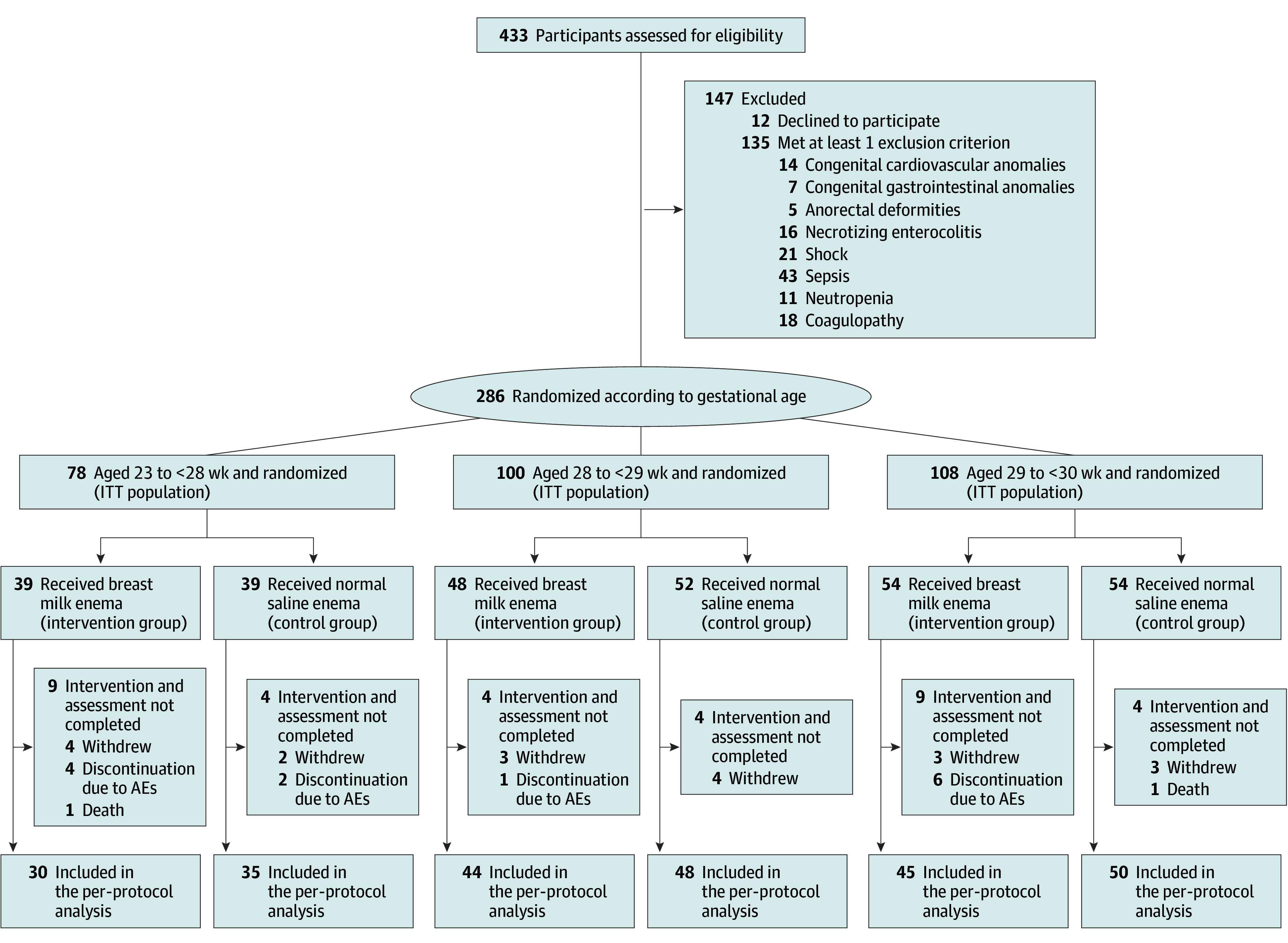
Study Flowchart for Screening, Randomization, and Follow-Up AE indicates adverse event; ITT, intention-to-treat.

The recruitment and retention of infants are shown in Figure 1. Among the 433 preterm infants initially screened, 12 parents declined to participate, and 135 infants met at least 1 exclusion criterion. Ultimately, 286 preterm infants (mean [SD] gestational age, 198.8 [7.9] days; 166 boys [58.0%] and 120 girls [42.0%]) with a mean (SD) birth weight of 1115.5 (237.8) g were eligible and included in this study (Table 1). A total of 145 infants (81 boys [55.9%] and 64 girls [44.1%]) were randomized to the normal saline group and 141 to the breast milk group (85 boys [60.3%] and 56 girls [39.7%]). The actual sample sizes at each gestational age were 78 for infants aged 23 to less than 28 weeks, 100 for infants aged 28 to less than 29 weeks, and 108 for infants aged 29 to less than 30 weeks. In the breast milk and normal saline groups, 22 and 12 preterm infants were lost to follow-up, respectively.

**Table 1.** Baseline Characteristics of Infants and Their Mothers^a^

Characteristic	All infants (N = 286)		Infants aged 23 to <28 wk (n = 78)		Infants aged 28 to <29 wk (n = 100)		Infants aged 29 to <30 wk (n = 108)	
	Normal saline (n = 145)	Breast milk (n = 141)	Normal saline (n = 39)	Breast milk (n = 39)	Normal saline (n = 52)	Breast milk (n = 48)	Normal saline (n = 54)	Breast milk (n = 54)
**Maternal characteristics**								
Mother’s age, mean (SD), y	32.2 (4.5)	32.0 (5.0)	32.7 (4.5)	30.9 (4.8)	31.8 (4.2)	31.3 (4.1)	32.2 (4.8)	33.4 (5.6)
Educational level, No. (%)								
Less than a high school diploma	39 (26.9)	30 (21.3)	9 (23.1)	8 (20.5)	17 (32.7)	8 (16.7)	13 (24.1)	14 (25.9)
High school degree	30 (20.7)	36 (25.5)	10 (25.6)	8 (20.5)	10 (19.2)	15 (31.3)	10 (18.5)	13 (24.1)
Associate’s degree	30 (20.7)	26 (18.4)	7 (17.9)	6 (15.4)	11 (21.2)	9 (18.8)	12 (22.2)	11 (20.4)
Bachelor’s degree and above	46 (31.7)	49 (34.8)	13 (33.3)	17 (43.6)	14 (26.9)	16 (33.3)	19 (35.2)	16 (29.6)
Cesarean delivery, No. (%)	91 (62.8)	86 (61.0)	22 (56.4)	17 (43.6)	32 (61.5)	32 (66.7)	37 (68.5)	37 (68.5)
Pregnancies, median (IQR), No.	2.0 (1.0-3.0)	2.0 (1.0-3.0)	2.0 (1.0-3.0)	2.0 (1.0-3.0)	2.0 (1.0-2.0)	2.0 (1.0-3.0)	2.0 (1.0-3.0)	2.0 (1.0-3.0)
Parity, median (IQR), No.	1.0 (1.0-2.0)	1.0 (1.0-2.0)	1.0 (1.0-2.0)	1.0 (1.0-2.0)	1.0 (1.0-2.0)	1.0 (1.0-2.0)	1.0 (1.0-2.0)	1.5 (1.0-2.0)
Pregnancy-induced hypertension, No. (%)	37 (25.5)	37 (26.2)	8 (20.5)	8 (20.5)	12 (23.1)	18 (37.5)	17 (31.5)	11 (20.4)
Diabetes, No. (%)	25 (17.2)	22 (15.6)	6 (15.4)	3 (7.7)	10 (19.2)	8 (18.8)	9 (16.7)	10 (18.5)
Medication use during pregnancy, No. (%)	62 (42.8)	78 (55.3)	17 (43.6)	22 (56.4)	21 (40.4)	31 (64.6)	24 (44.4)	25 (46.3)
**Infant characteristics**								
Infant sex, No. (%)								
Female	64 (44.1)	56 (39.7)	26 (66.7)	25 (64.1)	28 (53.8)	17 (35.4)	23 (42.6)	25 (46.3)
Male	81 (55.9)	85 (60.3)	13 (33.3)	14 (35.9)	24 (46.2)	31 (64.6)	31 (57.4)	29 (53.7)
Gestational age, mean (SD), d	198.6 (7.7)	198.9 (8.1)	189.5 (5.8)	188.1 (5.7)	198.8 (1.8)	199.3 (2.1)	205.8 (2.2)	206.3 (2.1)
Birth weight, mean (SD), g	1109.6 (219.9)	1121.5 (255.6)	953.9 (151.5)	928.2 (191.6)	1097.3 (207.2)	1119.8 (245.8)	1233.9 (199.6)	1262.6 (211.9)
Apgar score, median (IQR)								
1 min	7.0 (6.0-8.0)	7.0 (6.0-8.0)	6.0 (4.0-7.0)	6.0 (5.0-7.0)	7.0 (6.0-8.0)	7.0 (6.0-8.0)	8.0 (6.0-9.0)	7.0 (6.0-8.0)
5 min	9.0 (8.0-9.0)	9.0 (8.0-9.0)	8.0 (7.0-9.0)	8.0 (8.0-9.0)	9.0 (8.0-9.0)	8.0 (8.0-9.0)	9.0 (8.0-9.0)	9.0 (8.0-9.0)

^a^ Data include all patients in the full analysis set, unless indicated otherwise.

**Figure 2.**
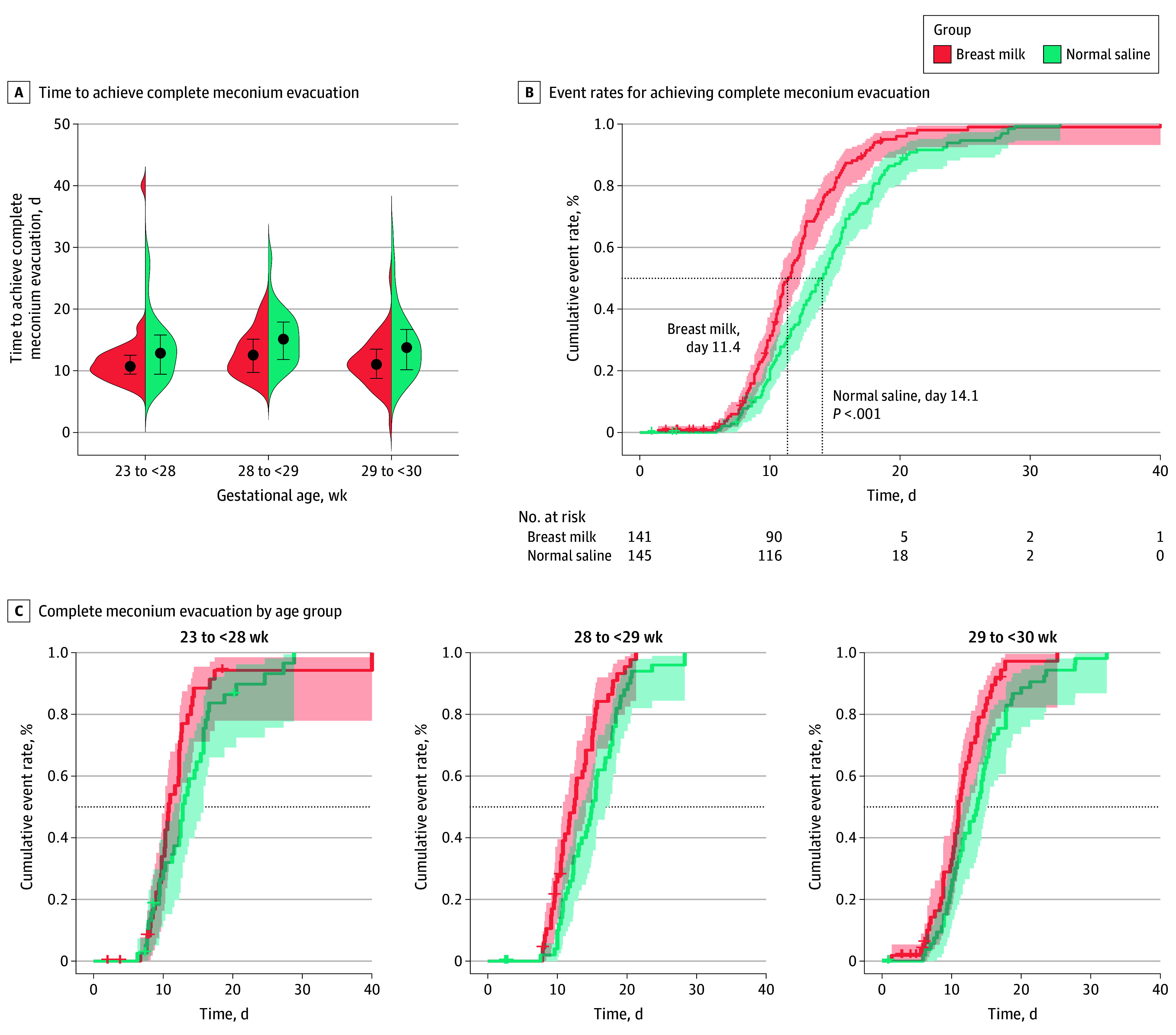
Time to Achieve Complete Meconium Evacuation A, Violin plots represent the entire range of the time to achieve complete meconium evacuation across 3 age subgroups for both the intervention and control groups. The dots represent median values, and lines represent the first and third quartiles. B, Kaplan-Meier estimates for event rates for achieving complete meconium evacuation in the intervention and control groups for all participants. The dotted horizontal line represents an event rate of 50%. The dotted vertical lines represent specific time points. The shaded areas represent 95% CIs. C, Subgroup analysis revealed that the breast milk group achieved complete meconium evacuation significantly faster than the normal saline group among infants aged 28 to less than 29 weeks and those aged 29 to less than 30 weeks. However, there was no significant difference among infants aged 23 to less than 28 weeks.

The sociodemographic characteristics, the clinical features of the mothers, birth anthropometric measures, infant sex, birth type, Apgar score, and gestational age at birth in the normal saline and breast milk groups are shown in Table 1. As shown in Table 2, the time to achieve complete meconium evacuation was significantly shorter in the breast milk group than in the normal saline group (11.4 days [IQR, 9.6-14.1 days] vs 13.7 [IQR, 10.6-16.9 days] days; difference, –2.2 days [95% CI, −3.2 to −1.2 days]; *P* < .001). Subgroup analysis showed sustained significance in the age groups of 28 to less than 29 weeks and 29 to less than 30 weeks (28 to <29 weeks: breast milk group, 12.5 days [IQR, 10.0-15.3 days]; normal saline group, 14.8 days [IQR, 11.5-17.9 days]; *P* = .008; 29 to <30 weeks: breast milk group, 11.0 days [IQR, 8.8-13.7 days]; normal saline group, 13.7 days [IQR, 10.3-17.8 days]; *P* = .01). However, the advantage in the breast milk group was not statistically significant for infants aged 23 to less than 28 weeks (breast milk, 10.7 days [IQR, 9.4-12.7 days] vs 12.8 days [IQR, 9.5-15.8 days]; *P* = .14). Figure 2A illustrates the distribution of the time to achieve complete meconium evacuation according to the gestational age subgroups. The median time to achieve complete meconium evacuation was calculated as the point at which 50% of the participants accomplished this outcome. The breast milk group exhibited a significantly shorter median time to complete meconium evacuation than the normal saline group (11.4 days [IQR, 9.6-14.1 days] vs 14.1 days [IQR, 10.7-17.6 days]; *P* < .001) (Figure 2B). This trend persisted among the infants aged 28 to less than 29 weeks (median, 12.4 days [IQR, 9.7-15.1 days] vs 15.0 days [IQR, 11.8-18.0 days]; *P* = .01) and among the infants aged 29 to less than 30 weeks (median, 11.0 days [IQR, 8.8-13.7 days] vs 13.7 [IQR, 10.1-16.7 days] days; *P* = .01), although not among the infants aged 23 to less than 28 weeks (Figure 2C).

**Table 2.** Effect of Breast Milk Enema on Primary and Secondary Outcomes Among Overall Participants and in Subgroup Analyses^a^

Outcome	All infants				Infants aged 23 to <28 wk				Infants aged 28 to <29 wk				Infants aged 29 to <30 wk			
	Normal saline, median (IQR)	Breast milk, median (IQR)	Estimated difference (95% CI)	*P* value	Normal saline, median (IQR)	Breast milk, median (IQR)	Estimated difference (95% CI)	*P* value	Normal saline, median (IQR)	Breast milk, median (IQR)	Estimated difference (95% CI)	*P* value	Normal saline, median (IQR)	Breast milk, median (IQR)	Estimated difference (95% CI)	*P* value
Time to achieve complete meconium evacuation, d	13.7 (10.6 to 16.9)	11.4 (9.6 to 14.1)	−2.2 (−3.2 to −1.2)	<.001	12.8 (9.5 to 15.8)	10.7 (9.4 to 12.7)	−1.8 (−3.5 to 0.2)	.14	14.8 (11.5 to 17.9)	12.5 (10.0 to 15.3)	−2.4 (−4.0 to −0.8)	.008	13.7 (10.3 to 17.8)	11.0 (8.8 to 13.7)	−2.4 (−4.0 to −0.6)	.01
Time to achieve full enteral feeding, d	35.4 (25.6 to 46.8)	29.5 (21.5 to 42.2)	−4.6 (−8.0 to −1.2)	.02	46.5 (31.9 to 65.0)	31.6 (24.7 to 48.7)	−10.1 (−18.6 to −1.7)	.03	35.5 (27.5 to 44.8)	31.6 (24.9 to 42.8)	−2.6 (−8.3 to 2.9)	.69	26.8 (21.8 to 36.7)	22.7 (18.7 to 32.8)	−4.1 (−8.3 to 0.1)	.10
Duration of TPN, d	35.8 (25.7 to 50.1)	30.5 (21.8 to 42.8)	−4.6 (−8.2 to −1.0)	.01	47.5 (34.0 to 65.0)	31.5 (24.1 to 49.7)	−10.4 (−19.1 to −2.2)	.01	35.5 (27.4 to 45.0)	32.9 (24.9 to 42.8)	−1.5 (−7.5 to 4.1)	.60	27.6 (21.7 to 39.6)	22.9 (18.8 to 36.0)	−4.3 (−9.0 to 0.2)	.06
Hospitalization for infant, d	63.5 (50.0 to 77.8)	62.0 (50.0 to 77.0)	−4.0 (−10.0 to 1.0)	.13	81.0 (68.0 to 93.0)	84.0 (73.0 to 94.0)	−5.0 (−17.0 to 6.0)	.41	65.0 (50.0 to 76.0)	64.0 (53.0 to 75.0)	−3.0 (−10.0 to 4.0)	.51	51.0 (44.8 to 60.8)	50.0 (42.5 to 61.0)	−3.0 (−9.0 to 3.0)	.27
Weight at discharge, g	2240.0 (1990.0 to 2557.5)	2270.0 (2020.0 to 2500.0)	0.0 (−90.0 to 90.0)	.94	2550.0 (2220.0 to 2850.0)	2400.0 (2100.0 to 2840.0)	−160.0 (−410.0 to 80.0)	.20	2140.0 (1940.0 to 2440.0)	2240.0 (2010.0 to 2525.0)	55.0 (−110.0 to 210.0)	.48	2165.0 (1957.5 to 2365.0)	2230.0 (2015.0 to 2400.0)	60.0 (−60.0 to 170.0)	.28

Abbreviation: TPN, total parenteral nutrition.

^a^ The median difference and 95% CIs were assessed using the Mann-Whitney test and the Hodges-Lehmann method.

The time to achieve full enteral feeding was also significantly shorter in the breast milk group than in the normal saline group for all participants (29.5 days [IQR, 21.5-42.2 days] vs 35.4 days [IQR, 25.6-46.8 days]; *P* = .02) and for infants aged 23 to less than 28 weeks (31.6 days [IQR, 24.7-48.7 days] vs 46.5 days [IQR, 31.9-65.0 days]; *P* = .03) (Table 2). Breast milk enemas facilitated total enteral feeding 4.6 days (95% CI, −8.0 to −1.2 days) earlier for all participants and 10.1 days (95% CI, –18.6 to –1.7 days) earlier for infants aged 23 to less than 28 weeks. Among infants aged 28 to less than 29 weeks, the intervention and control groups achieved full enteral feeding in 31.6 days (IQR, 24.9-42.8 days) and 35.5 days (IQR, 27.5-44.8 days), respectively (*P* = .69). Among infants aged 29 to less than 30 weeks, the intervention and control groups achieved full enteral feeding in 22.7 days (IQR, 18.7-32.8 days) and 26.8 days (IQR, 21.8-36.7 days), respectively (*P* = .10). Although both intervention groups experienced a reduction, the difference was not statistically significant. However, the observed differences may still be clinically significant.

**Figure 3.**
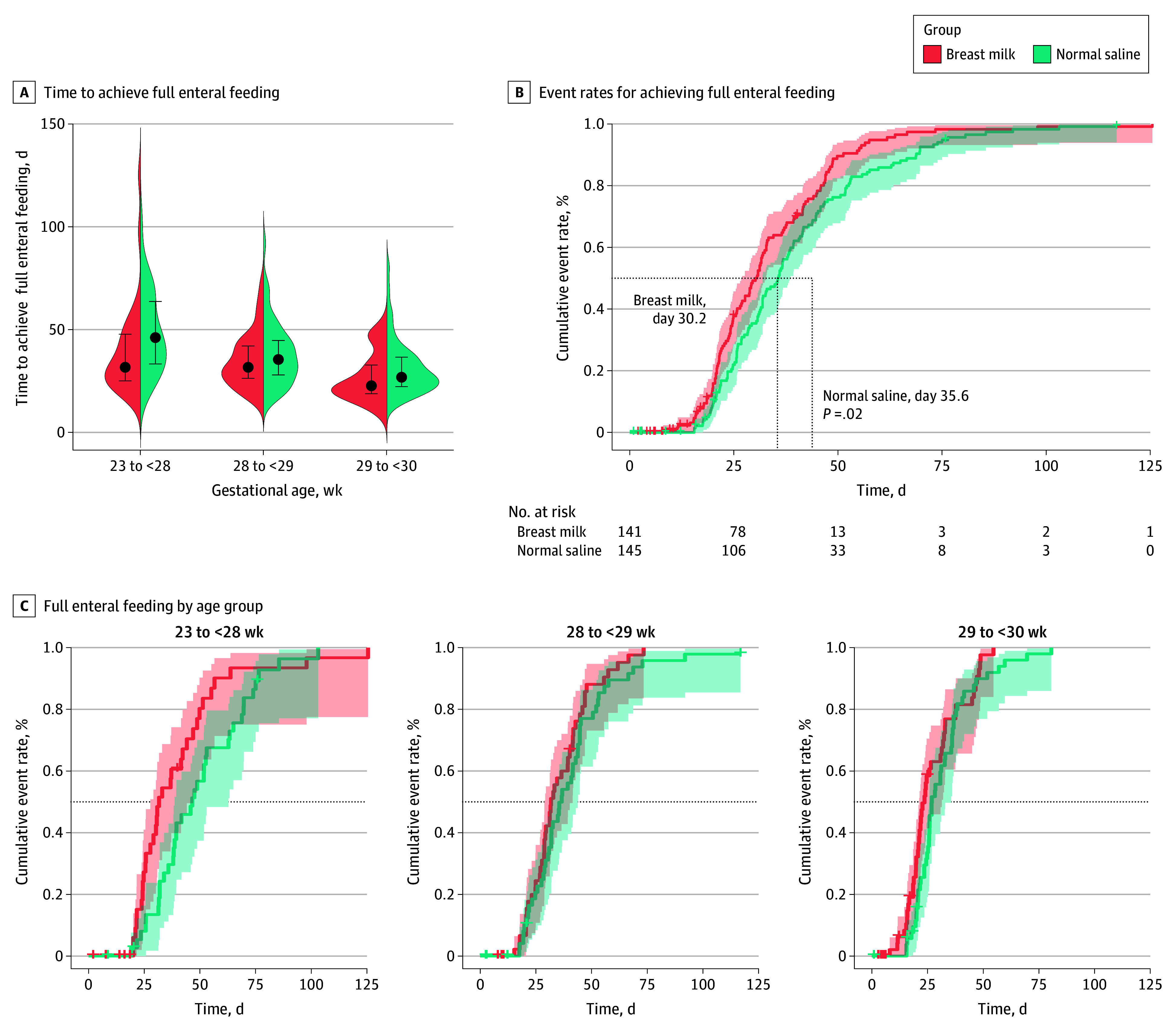
Time to Achieve Full Enteral Feeding A, Violin plots represent the entire range of the time to achieve full enteral feeding across 3 age subgroups for both the intervention and control groups. The dots represent median values, and lines represent the first and third quartiles. B, Kaplan-Meier estimates for event rates for achieving full enteral feeding in the intervention and control groups for all participants. The dotted horizontal line represents an event rate of 50%. The dotted vertical lines represent specific time points. The shaded areas represent 95% CIs. The breast milk group exhibited a significantly shorter median time to achieve full enteral feeding compared with the normal saline group. C, Subgroup analysis revealed no significant difference among the 3 subgroups.

Violin plots in Figure 3A indicate the distribution of time to achieve full enteral feeding according to the gestational age subgroups. Similar to previous analyses, for all participants, the breast milk group exhibited a significantly shorter median time to achieve full enteral feeding compared with the normal saline group (30.2 days [IQR, 21.6-42.8 days] vs 35.6 days [IQR, 25.7-47.5 days]; *P* = .02) (Figure 3B). This finding suggests that the use of breast milk enemas accelerates the attainment of both complete meconium evacuation and full enteral feeding.

As for secondary outcomes, the duration of TPN was found to be significantly shorter in the breast milk group than in the normal saline group for all participants (30.5 days [IQR, 21.8-42.8 days] vs 35.8 days [IQR, 25.7-50.1 days]; difference, −4.6 days [95% CI, −8.6 to −1.0 days]; *P* = .01) and among infants aged 23 to less than 28 weeks (31.5 days [IQR, 24.1-49.7 days] vs 47.5 days [IQR, 34.0-65.0 days]; *P* = .01); however, no significant differences were observed among infants aged 28 to less than 29 weeks and those aged 29 to less than 30 weeks (Table 2). Moreover, neither the total number of participants nor subgroup analyses revealed statistically significant differences in duration of hospitalization for infants or weight at discharge. The median duration of hospitalization for the intervention and control groups were 62.0 days (IQR, 50.0-77.0 days) and 63.5 days (IQR, 50.0-77.8 days), respectively (*P* = .13). The median infant weight at discharge in the intervention and control groups were 2270 g (IQR, 2020-2500 g) and 2240 g (IQR, 1990-2558 g), respectively (*P* = .94).

In the safety analysis, no significant differences were observed between the intervention and control groups across all safety outcomes, including bronchopulmonary dysplasia, late-onset sepsis, retinopathy of prematurity, intraventricular hemorrhage, NEC, bloody stools, and mortality. These findings remained consistent in subgroup analyses, as presented in eTable 1 in Supplement 2. However, all 4 cases of colorectal and anal injuries occurred in the breast milk group and were confined to infants aged 29 to less than 30 weeks’ gestational age.

Sensitivity analyses were generally consistent with the main analysis (eTable 2 and eTable 3 in Supplement 2). In addition to the intention-to-treat analysis, a reanalysis using the per-protocol dataset yielded results consistent with the main findings, with no statistically significant disparities (eTable 4, eTable 5, and eTable 6 in Supplement 2).

## Discussion

This RCT compared the administration of breast milk enemas with that of normal saline enemas among preterm infants and revealed significant benefits. The breast milk group exhibited a shorter time to achieve complete meconium evacuation, faster attainment of full enteral feeding, and a reduced duration of TPN. Various methods have been used in routine neonatal care to promote meconium evacuation; however, a literature review indicates that there is no consensus on the agents used and the frequency of applications. To our knowledge, this is the first study to use breast milk as an enema agent, representing a novel approach.

Our findings differed from a prior investigation by de Pipaón Marcos et al,^1^ which used rectal stimulation and normal saline enemas. Varied definitions of complete meconium evacuation contributed to a longer observed duration in our study than in the study by de Pipaón Marcos et al^1^ (10.7 vs 9 days). Specifically, we defined this period as the time until the occurrence of 1 day of yellow stool, covering both complete evacuation of the meconium and transition stools. Our study also deviates from the findings by de Pipaón Marcos et al^1^ regarding the time to achieve full enteral feeding (31.6 vs 16 days). Discrepancies may arise from variations in the definition of full enteral feeding; we used 180 mL/kg/d and weight gain more than 20 to 25 g/d within 24 hours, while the criteria in the study by de Pipaón Marcos et al^1^ were 120 mL/kg/d for full enteral feeding. Furthermore, our feeding approach was cautious, whereby feeding was halted when the residual gastric volume surpassed half of the feeding volume. This strategy was guided by a comprehensive evaluation^26^ that highlighted gastric residual volume as the predominant factor impeding the progression of feeding. Another study^27^ further supported this perspective and indicated that routine evaluation of residual gastric volume prolonged the attainment of full enteral feeding and the duration of TPN.

Subgroup analyses discerned nuanced differences in the intervention’s efficacy among different gestational age subgroups, providing a more detailed insight into its effect. Primarily, the time to achieve complete meconium evacuation showed statistical significance only among infants aged 28 to less than 29 weeks and those aged 29 to less than 30 weeks. This discrepancy may stem from the heterogeneous developmental trajectories in the gastrointestinal milieu of preterm neonates.^28^ For the relatively more mature subgroup of infants aged 28 to less than 30 weeks, breast milk enemas are postulated to augment gastrointestinal motility, expediting meconium evacuation. Conversely, neonates aged 23 to less than 28 weeks might require a more nuanced and tailored stimulus given their relatively nascent gastrointestinal milieu. This finding emphasizes the need to consider the effectiveness of the intervention, considering the distinctive physiological variations present among preterm infants across different gestational age categories. Conversely, the time to achieve full enteral feeding followed a different trajectory. A significant intervention effect was found only among infants aged 23 to less than 28 weeks. This differential response could reflect the disparate effect of the intervention on feeding tolerance and gastrointestinal maturation across diverse gestational age cohorts.^29^ Among infants aged 23 to less than 28 weeks, breast milk enemas likely enhanced feeding tolerance by promoting gastrointestinal motility and augmenting maturation for a quicker transition to comprehensive enteral nutrition. However, among infants aged 28 to less than 30 weeks, for whom gastrointestinal functionality approached maturation, the effects of the intervention may have dwindled.

Human milk is a complex biological substance containing not only macronutrients and micronutrients but also living cells, growth factors, and immunoprotective substances.^30,31^ Many of these factors are resistant to digestive enzymes in the infant gastrointestinal tract and are biologically active on the mucosal surfaces, stimulating gastrointestinal growth and motility. Breast milk is a natural oil-in-water emulsion,^32^ which can soften meconium to a certain extent and lubricate and stimulate the intestinal wall, making it easier to excrete the meconium. Simultaneously, oligosaccharides in human breast milk can promote the colonization of probiotics in the intestinal tract of newborns,^33^ contribute to the integrity of the gastrointestinal epithelial barrier in preterm infants, promote intestinal maturation,^34^ enhance feeding tolerance, and achieve total enteral feeding as soon as possible. Although evidence suggests that breast milk can improve the immunity of preterm infants,^35^ there was no significant difference in most safety outcomes, except colorectal and anal injuries between the normal saline and breast milk groups in all age subgroups. Consequently, the confirmed safety of breast milk enemas remains noteworthy. Future studies are warranted to further explore and highlight the necessity for continued safety monitoring.

### Strengths and Limitations

Our study has several strengths. It stands as the first RCT to investigate the efficacy and safety of breast milk as an enema agent, providing robust support for this novel approach. The use of an RCT design enhances objectivity and credibility in drawing conclusions. In addition, this study innovatively categorizes preterm infants according to gestational age. Furthermore, to our knowledge, our sample size on facilitating meconium excretion is the largest to date, allowing for stratified analyses by gestational age and thereby augmenting statistical power.

However, our study also has some limitations. First, stratified analysis based on birth weight was not conducted. Nonetheless, the results from the stratified analysis based on gestational age remained consistent with the overall analysis. Second, this study was limited to a single center, thereby reducing the generalizability of its findings compared with multicenter studies.

## Conclusion

This RCT found that, in comparison with normal saline enemas, breast milk enemas have been demonstrated to be both effective and safe, resulting in a reduction in the time to achieve complete meconium evacuation, time to achieve full enteral feeding, and duration of TPN among preterm infants. Subgroup analyses highlight the complex nature of neonatal development, emphasizing the need for tailored interventions based on gestational age considerations. To corroborate our findings further, a multicenter RCT is crucial, providing broader insights into the potential benefits of breast milk enemas for preterm infant care.

## Notes

Supplement 1.
Trial Protocol and Statistical Analysis Plan

Supplement 2. **eTable 1.** Safety Outcomes Among Overall Participants and in Subgroup Analyses for the ITT Set **eTable 2.** Sensitivity Analyses on Primary Outcomes **eTable 3.** Adverse Events Leading to Discontinuation Among All Participants **eTable 4.** Baseline Characteristics of Infants and Their Mothers **eTable 5.** Effect of Breast Milk Enema on Primary and Secondary Outcomes Among Overall Participants and in Subgroup Analyses **eTable 6.** Safety Outcomes Among Overall Participants and in Subgroup Analyses for the PP Set

Supplement 3.
Data Sharing Statement
