# Supplementary material for: Breast Milk Enema and Meconium Evacuation Among Preterm Infants: A Randomized Clinical Trial
Source: JAMA Netw Open. 2024 Apr 22;7(4):e247145. doi: 10.1001/jamanetworkopen.2024.7145 (PMC11981638; doi:10.1001/jamanetworkopen.2024.7145)
Supplement: Supplement 1. — Trial Protocol and Statistical Analysis Plan [file jamanetwopen-e247145-s001.pdf]

CLINICAL STUDY PROTOCOL

Clinical Effects of Breast Milk on Enema-induced Meconium Evacuation in Super Premature Infants and Preterm Infants: A Randomized Controlled Trial

PROTOCOL VERSION 2.0

Dated: February 20, 2019

Shenyang, China

Table of Contents

1 Background **4**

2 Objectives **5**

3 Study design and organization **5**

3.1 Overview of the study design 5

3.2 Study organization 6

4 Study population **7**

4.1 Recruiting criteria 7

4.2 Recruitment 8

4.3 Informed consent 9

4.4 Additional consent provisions 9

5 Randomization **9**

6 Blinding **10**

7 Intervention **10**

7.1 Intervention scheme 10

7.2 Feeding schedule 11

7.3 Study termination 11

8 Study end-points **12**

8.1 Primary end-points 12

8.2 Secondary end-points 12

9 Data collection **12**

9.1 Baseline demographic information 13

9.2 Follow-up data collection 13

10 Data management and quality control **13**

10.1 Baseline data quality control 14

10.2 Follow-up data quality control 14

10.3 Missing data 14

11 Sample size and statistical power estimation **14**

12 Study Management **15**

12.1 Oversight and monitoring 15
12.2 Adverse event reporting and harms 15
12.3 Frequency and plans for auditing trial conduct....................................16
12.4 Plans for communicating important protocol amendments to relevant parties 16
12.5 Dissemination plans 16
12.6 Biological specimens 16
12.7 Confidentiality 16
12.8 Study schedule 16
13 Ethics approval and consent to participate 17
14 Statistical analysis plan 17
14.1Descriptive statistics and baseline characteristics.................................17
14.2 Analysis of primary outcomes 17
14.3 Analysis of secondary outcomes...18
14.4 Analysis of safety..18
14.5 Subgroup analysis 18
14.6 Statistical software 19
15 Discussion..19
References...21

1. Background

According to estimates, 15 million premature births occur every year worldwide[1]. China has the second largest number of premature infants in the world, 1.17 million premature births are reported in China every year, accounting for 10% of all newborns[2]. Delayed action of full enteral nutrition in preterm infants is closely associated with an increase in morbidity and mortality[3-5]. Delayed meconium evacuation is a recognized cause of intestinal dysfunction[6-7], which can cause delayed feeding, gastric retention, and feeding intolerancein preterm infants. The immature intestinal motility mechanism and neurotransmitter system of low-birth-weight infants delay meconium evacuation. Moreover, viscous meconium causes functional blockage of the intestines and abnormal gastrointestinal functions. The younger the gestational age of premature infants, the later and longer the meconium evacuation[8-11]. Studies have shown that early meconium evacuation improves feeding intolerance and promotes weight gain[12]. Because the meconium contains high levels of bilirubin, a delay in meconium evacuation increases the intestinal circulation of bilirubin, thereby aggravating neonatal jaundice and increasing the risk of bilirubin brain damage, kernicterus, serious sequelae, and even death[13].

Currently, the widely used strategies for inducing meconium evacuation worldwide are glass rod stimulation of the rectum, enema with formulations and oral intake of glucosamine (Gastrografin)[14-17]. Among them, enemas are the most commonly used approach. The formulations used for enemas include solutions (including physiological saline, glycerin, and diatrizoate) and suppositories (mainly glycerin). Glucosamine, a formulation for meconium evacuation that has emerged in recent years, is a gastrointestinal contrast agent that can be administered orally or rectally and has a hyperosmotic effect[17]. However, the effects of glucosamine in meconium evacuation remain controversial[10]. Glycerin solutions and glycerol suppositories have been widely used abroad as enema formulations, although a meta-analysis has suggested that the effects of glycerol solutions or glycerol suppositories in preterm

infants are unclear[18]. Saline is the most widely used formulation, but a systematic review has shown that saline does not shorten the total feeding time of preterm infants[19].

Because the effects of the above commonly used enema formulations are controversial, identifying a new enema formulation is necessary to increase meconium evacuation in premature infants. Studies have shown that breast milk has appropriate osmotic pressure and high safety in stimulating the digestive tract in infants[20]. Therefore, we aimed to explore whether a breast milk based enema might shorten the time of the last meconium evacuation and the time to achieve full enteral feeding in super premature infants and preterm infants.

2. Objectives

(1) Explore whether breast milk can shorten the time of the last meconium evacuation in super premature infants and preterm infants.

(2) Explore whether breast milk can shorten the time of full enteral feeding in super premature infants and preterm infants.

(3) Evaluate whether breast milk can decrease the enterohepatic circulation of bilirubin and the incidence of cholestasisin super premature infants and preterm infants.

3. Study design and organization

3.1 Overview of the study design

We aim to explore whether breast milk might shorten the time of the last meconium evacuation and the time of achieve full enteral feeding in super premature and preterm infants. Saline will be used as a control. We will use a randomized controlled trial to recruit eligible subjects and assign them to intervention and control groups. The control group will receive saline, and the intervention group will receive breast milk for meconium evacuation (**Figure 1**).

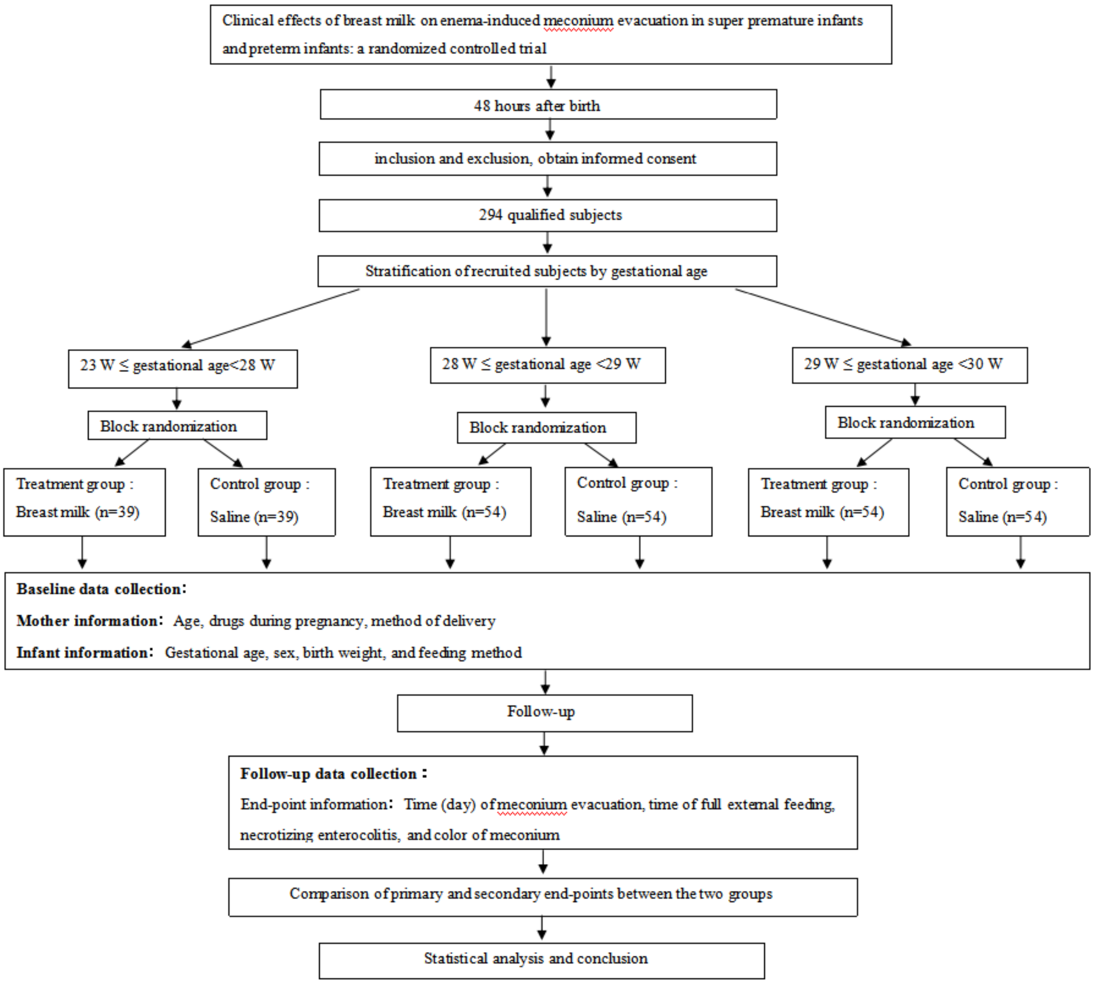

Figure 1. **Flow chart of study design.**

3.2 Study organization

Figure 2 shows the study organization.

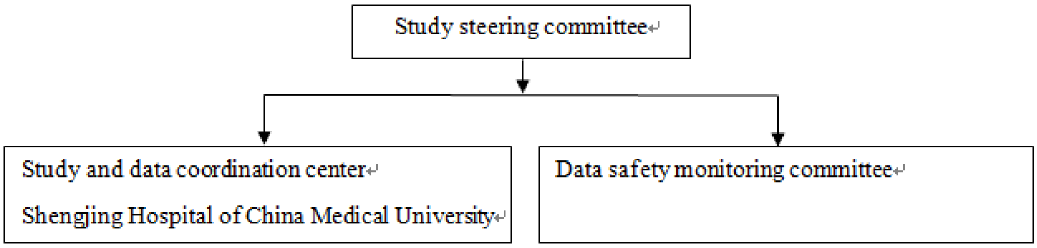

Figure 2. Study organization.

3.2.1 Study steering committee

This trial will be supervised by the study steering committee, which will consist of experts in epidemiology, pediatrics, and infectious disease. The committee will oversee the study design, implementation, data analysis, and reporting of the trial. The main responsibilities of the committee will include the execution of research programs, personnel training, supervision, and guidance.

3.2.2 Study and data coordination center

The study and data coordination center is located at Shengjing Hospital of China Medical University. The study and data coordination center will work closely with the study steering committee to provide scientific and administrative support for the trial. The main responsibilities of the study and data coordination center will include implementation of the randomization process, data quality control, preparation of reports, and data analysis for the study steering committee and the data safety monitoring committee. In addition, it will meet weekly to communicate issues and perform troubleshooting.

3.2.3 Data safety monitoring committee

The data safety monitoring committee will monitor any safety issues that occur during the trial, and will conduct monitoring and recording and propose solutions.

4. Study population

4.1 Recruiting criteria

In this project, we will recruit super premature infants and preterm infants at the Department of Neonatology, Shengjing Hospital of China Medical University as eligible subjects. The inclusion and exclusion criteria for the study participants will be as follows:

Inclusion criteria:

(1) Super premature infants 23W ≤ gestational age <28W

Preterm infants 28W ≤ gestational age <30W

(2) Mother provides breast milk 48 hours after birth.

(3) Normal intestinal motility (as assessed by the team of clinical experts)

(4) Parental agreement to participate in the study

Exclusion criteria:

Premature infants meeting any of the following table will be excluded.

**Criteria**	**Definition**
Congenital malformations	Congenital malformations are structural or chromosomal malformations that have a major impact on health and development.
Congenital gastrointestinal anomalies	Congenital gastrointestinal anomalies are mainly diagnosed by ultrasonography or radiography. Congenital gastrointestinal anomalies include gastric volvulus, cecal volvulus and heterotopic pancreas, etc.
Anorectal deformities	Anorectal deformities are a wide spectrum of abnormalities of the anus and rectum, which include anal atresia, rectovesical fistula, etc. Anorectal deformities will be diagnosed by Magnetic resonance or sonography.
Diarrhea	Diarrhoea is defined as the occurrence of 3 or more loose stools within 24h.
Intussusception	Intussusception is characterized by telescoping of one part of the gastrointestinal tract into another part, forming an obstruction. Intussusception will be diagnosed by ultrasound.
NEC	The main symptoms of NEC are abdominal distension and hematochezia, which are characterized by intestinal mucosal necrosis. NEC is mainly diagnosed by abdominal X-ray and ultrasoud examiantion.
PDA	PDA will be confirmed by echocardiography
Sepsis	Sepsis includes early-onset and late-onset. Early-onset sepsis is caused by pathogens transmitted from mothers, and late-onset sepsis is caused by nosocomial infections. Sepsis is defined as the overgrowth of bacteria in blood cultures in the presence of clinical deterioration.
Neutropenia	Absolute neutrophil count $<0.5\times10^{9}$/L
Coagulopathy	International standardization ratio >1.4, Partial thromboplastin time >39s, Fibrinogen <1.00g/L, Platelet count $<100\times10^{9}$/L

Necrotizing enterocolitis: NEC ; Patent ductus arteriosus: PDA.

4.2 Recruitment

We propose to recruit 78 super premature infants and 216 preterm infants from the Department of Neonatology, Shengjing Hospital of China Medical University. Recruitment will occur from June 2019 to September 2020 and will be conducted on-site. Leaflets with trial information will be displayed onsite in the waiting room of the neonatology department. A research assistant will ask each participant's guardian if they would be interested in having their babies participate and will be given sufficient time to make their decision. The guardian can contact the research assistant using the provided telephone number and undergo screening to enter the trial.

4.3 Informed consent

If the guardian is willing and the infant is eligible to participate in the study, the participant's guardian will be required to sign a written informed consent. Informed consent procedures will ensure that participant's guardian understands participation is voluntary and that participants can withdraw from the study at any time. The informed consent can be obtained from the corresponding author.

4.4 Additional consent provisions

The main trial consent asks mothers of participants if they would be willing to allow the use of their baby's data if they choose to withdraw from the trial. This trial does not involve collecting biological specimens for storage.

5. Randomization

Participants will be allocated to intervention and control groups with stratified block randomization using a computer-generated randomization list by an independent statistician not otherwise involved in the trial. The participants will be stratified based on gestational age. Then, the participants will be assigned in a randomized block design to the intervention and control groups in a 1:1 ratio. Block size will be 4 and 6 to ensure equal numbers of participants in the intervention and control groups. At the beginning of the trial, random sequence will generate a set of study numbers. Each study number will be paired with a method of intervention and sealed in an opaque envelope. The envelopes will be grouped based on gestational age. Recruited

participants will be allocated to intervention or control groups by selecting an envelope. The program will be set up using SAS 9.2 (SAS Institute Inc., Cary, NC).

6. Blinding

The design is open-label with only outcome assessors and data analysts being blinded to decrease bias and increase the accuracy of data collection.

7. Intervention

7.1 Intervention scheme

Preterm infants in control and intervention groups will receive a saline and breast milk enema, respectively. Nurses will be trained to ensure the standardized and safe administration of enemas. The timeline of the trial is shown in Figure 2. The specific procedures are described as follows:

(1) Meconium evacuation procedure: preterm infants in intervention and control groups will undergo meconium evacuation twice per day at 9:00 and 21:00. The intervention will continue until the meconium is completely evacuated.

(2) Preparation of materials: silicone tube (4–6 cm, model 3.33 mm [F10]), 5 mL syringe, thermostat (temperature setting 37°C), sesame oil, sterile gloves.

(3) Implementation: The number of enemas required will be calculated (5 mL/kg) and preheated to 37°C in a thermostatically controlled water bath. The preheated enemas will be extracted with a syringe. One end of the silicone tube will be connected to the syringe and the other end will be lubricated with sesame oil before inserting it 2–3 cm into the rectum. The enema will be slowly injected into the rectum and will be retained there for 3 minutes; the silicone tube will then slowly be pulled out.

If a premature infant does not pass stools during the 24 hours following the complete evacuation of meconium, an enema will be administered. A saline enema will be used once a day in both intervention and control groups using the same method as described above.

Criteria for the termination of intervention will include complete meconium evacuation, the development of any of the serious adverse events listed in the outcomes below or the withdrawal of a guardian's consent.

To improve adherence and promote participant retention, nurses and doctors will be trained in study-specific procedures prior to participation in the study. In addition, guardians will be updated weekly on the status of their infants by a nurse who is otherwise not involved in the study. The Trial Steering Committee (TSC) will also dynamically monitor the rate of follow-up loss.

7.2 Feeding schedule

The same feeding and parenteral nutrition procedures will be used for premature infants in control and intervention groups. The total daily calories of enteral and parenteral nutrition will be 110–135 kcal/kg. Premature infants will be fed starting at 24 mL/kg birthweight per day. Furthermore, feedings will be advanced every 24 hours by 20 mL/kg birthweight per day. When the amount of enteral feeding reaches 120 mL/kg per day, parenteral nutrition will be stopped. We defined full enteral feeding as tolerance of enteral feeding volumes of 180 mL/kg/day within 24 hours and a weight gain greater than 20–25 g/d within 24 hours. Human milk feeding will be encouraged. If the amount of breast milk is insufficient to meet the nutritional needs of premature infants, mixed feeding (artificial and breast milks) will be adopted. We will use disposable bottles for feeding to calculate the volume of feeding and use nasogastric feeding for premature babies with poor sucking ability. Feeding will be stopped if feeding intolerance occurs, such as when a gastric residual volume occurs that is more than half of the feeding volume, an abdominal circumference that is increased by 2 cm more than before the previous feeding, or with the occurrence of an intestinal dynamic anomaly.

7.3 Study termination

The study will be terminated in the event of sepsis, NEC, fecal occult blood positive, diarrhea, or rectal or anal injury.

8. Study end-points

8.1 Primary end-points

(1) Time from birth to the last meconium evacuation

This trial will use the standard infant meconium evacuation form. Nursing personnel will be trained to correctly identify the infant's meconium, transitional meconium, and normal feces. Detailed information on the evacuation and characteristics of each meconium will be recorded, and questionable meconium photos will be recorded and discussed in group meetings.

(2) Time to achieve total enteral feeding in super premature infants and preterm infants (when the infant tolerates a volume of 180 ml/kg/day for at least 24 h or undergoes weight gain greater than 20-25 g/d in 24 h)

8.2 Secondary end-points

(1) Stage II or III necrotizing enterocolitis (NEC, Bell standard)[21]

(2) Hospitalization days

(3) Body weight at discharge

(4) Duration of total parenteral nutrition

(5) Cholestasis[22](Serum binding bilirubin concentration > 1.0 mg/dL (17.1 mmol/L)

Serum total bilirubin < 5.0 mg/dL (85.5 mmol/L) or >20%

Total bilirubin concentration >5.0 mg/dL (85.5 mmol/L), measured 2 weeks after birth in two groups

(6) Any adverse events reported: nosocomial infection, nosocomial death, retinopathy of prematurity (ROP, any stage)[23], chronic lung disease (CLD), requirement of ventilatory support or oxygen after a corrected gestational age of 36 weeks [22], intraventricular hemorrhage (IVH, grade 2 and above)[24], bronchopulmonary dysplasia (BPD), late-onset sepsis (LOS), diarrhea, colon perforation, malabsorption, rectal bleeding, and rectal trauma.

9. Data collection

The research data will be collected by trained researchers using standardized questionnaires and measurements to ensure accuracy. At baseline, the following information regarding premature infants and their mothers will be collected: gestational age, birth weight, demographic information, history of disease, and medications used during pregnancy. The following major aspects of outcome information will be collected during the follow-up: meconium evacuation, feeding, laboratory tests, and adverse events.

9.1 Baseline demographic information

(1) Mother’s information: general information (name, age, date of birth, ethnicity, education level, ID number, home address, and contact information), medical history (pregnancy-induced hypertension syndrome and gestational diabetes), use of medicine during pregnancy, infant delivery method, and maternity.

(2) Infant’s information: gestational age, sex, birth weight (g), starting time of enteral feeding within 48 hours after birth, method of enteral feeding (breast milk only/breast milk formula mix/formula only), and Apgar score (1 min, 5min).

9.2 Follow-up data collection

(1) End-point information: body weight (g), meconium evacuation time (h), fecal type, abnormal traits and color; time to achieve total enteral feeding (h), duration of total parenteral nutrition (h), feeding tolerance (residual milk volume, nature of the residual liquid, abdominal circumference before the two feedings, bowel sounds, and intestinal motility conditions), route and method (breast milk only/breast milk formula mixed/formula milk powder only) of feeding, type of formula, C-reactive protein measurements, blood culture results, blood oxygen measurements, Whether to perform phototherapy, serum bilirubin levels, neonatal intensive care unit time, and NEC.

(2) Adverse events: nosocomial infection, in-hospital death, ROP, CLD, IVH, BPD, LOS, diarrhea, colon perforation, malabsorption, rectal bleeding, and rectal trauma.

10. Data management and quality control

All super premature and preterm infants will be uniquely coded (ID) according to the same rules. After the data are checked by the quality control commissioner, the Epidata database will be used for double entry. Logical verification and positioning and modification will be performed. The data will be managed throughout the process. The locked database will be used for analysis after the accuracy is confirmed.

10.1 Baseline data quality control

a. Carefully design and develop the questionnaire.

b. Consistently train investigators.

c. Implement consistent standards for all operations.

10.2 Follow-up data quality control

a. The infants will be monitored by dedicated nurses, and the treatment will be performed by a dedicated nurse.

b. Recorded events will be collected through consistent standards.

c. Integrated data entry and input verification will be performed.

10.3 Missing data

The relevant researcher will contact the premature infant's guardian by telephone or check electronic medical records to supplement missing baseline and follow-up data. If no reply is received from the guardian or missing data cannot be supplied, the missing data will be processed using different methods based on the mechanism of the missing data. For example, mean value imputation and last observation carried forward will be used to supplement the data. Furthermore, we will conduct a sensitivity analysis to evaluate the robustness of trial results: When we analyze primary outcomes, we will conduct a comparative analysis of complete (ignoring the missing data) and incomplete (no missing data) cases.

11. Sample size and statistical power estimation

The sample size estimate is based on our pilot study; the time of complete meconium evacuation was 1.4 days, 1.2 days, and 1.2 days earlier in the breast milk enema group than in the saline enema group at 23 w ⩽ gestational age < 28 w, 28 w ⩽

gestational age < 29 w, and 29 w ≤ gestational age < 30 w, respectively. Given that $\alpha = 0.05$, $\beta = 0.2$, standard deviation (SD) = 2, sampling ratio = 1 and taking a 20% drop rate into account, the required total sample size is 78, 108, and 108 premature infants in each gestational age, respectively. A website was used to calculate the sample size (http://powerandsamplesize.com/Calculators/).

Gestational age	Difference	α	β	SD	Sample size	Loss of follow-up	Final sample size
23W≤Gastinal age < 28W	1.4d	0.05	0.2	2	64	20%	78
28W≤Gastinal age < 29W	1.2d	0.05	0.2	2	88	20%	108
29W≤Gastinal age < 30W	1.2d	0.05	0.2	2	88	20%	108

12. Study Management

12.1 Oversight and monitoring

Composition of the coordinating center and trial steering committee and composition of the data monitoring committee, its role and reporting structure

This trial has been supervised by the TSC, which consists of experts in epidemiology, pediatrics, and infectious diseases. The main responsibilities of the committee include the supervision of study design, implementation, data analysis, and trial reports. The TSC will meet every 3 months. The Data Coordination Center (DCC) will mainly be responsible for the quality control of data and the implementation of randomization. The Data Security Monitoring Committee (DSMC) will monitor and record any security issues that occur with regard to participants during the trial and propose solutions. The DCC and DSMC will meet once a month. Furthermore, the DCC and DSMC will report to the TSC. Stakeholder and public involvement groups will not be permitted.

12.2 Adverse event reporting and harms

All adverse events observed by the investigators will be recorded and reported to the DSMC, highlighting any seriousness as required. Furthermore, any causality between intervention and adverse events will be recorded.

12.3 Frequency and plans for auditing trial conduct

The Trial Management Team will meet every 3 months to review the implementation of the study. The independent monitor will make an on-site visit once a month and check the quality of the data, including inclusion and exclusion criteria, informed consent, original data, and the absence of data.

12.4 Plans for communicating important protocol amendments to relevant parties

A substantial amendment is defined as an amendment to the protocol that is likely to have a significant effect on the safety of participants. Notification of all substantial amendments will be provided to the participants' guardians and ethics committees. Non-substantial amendments and any deviations from the protocol will be fully recorded using a breach report form. In addition, online trial registries will be updated accordingly.

12.5 Dissemination plans

The results of the study will be completely reported in international peer-reviewed journals. Both positive and negative results will be disclosed. We hope that our findings provide a potential clinical significance.

12.6 Biological specimens

Biological specimens will not be collected in this trial.

12.7 Confidentiality

During the course of the study, data collected will be kept strictly confidential and only accessed by members of the trial team. Data will be stored in a secure database. In publications, no identifying details of participants will be reported. We will determine whether anonymized trial data can be used for meta-analyses based on discussion by the ethics committee.

12.8 Study schedule

The project is proposed to be performed from June 2019 to September 2020. The researchers will follow the subjects daily until the study end-points are obtained or the trial must be terminated.

13. Ethics approval and consent to participate

The Shengjing Hospital of China Medical University Medical Ethics Committee has approved the research plan and written informed consent has been formally obtained from all participants' guardians during screening. The research protocol was approved by the Shengjing Hospital of China Medical University Medical Ethics Committee (No. 2019PS503K).

14. Statistical analysis plan

14.1 Descriptive statistics and baseline characteristics

We will conduct intention-to-treat (ITT) and per-protocol (PP) analyses. Descriptive statistics for all baseline characteristics will be reported for each group. Categorical variables will be presented as counts and percentages (%), while continuous variables will be presented as mean ± SD or medians with quartiles (Q1-Q3). The baseline data include demographic information, pregnancy-related details such as the occurrence of cesarean section, the number of pregnancies and parity, history of a mother's disease, medication history, as well as the newborn's gender, birth weight, and Apgar scores.

14.2 Analysis of Primary Outcomes

The two primary outcomes include the time to achieve complete meconium evacuation and the time to complete enteral feeding. Outcomes are presented as median (Q1-Q3).The outcomes will be analyzed using an independent sample t-test or Mann–Whitney test. Meanwhile, a multiple linear regression model will be used to analyze the factors affecting primary outcomes, including gender, birth weight, history of a mother's disease, etc. For outcome differences between the treatment and control arms, the median difference and 95% confidence interval (CI) will be assessed using the Mann–Whitney U test and the Hodges–Lehmann method. The time to achieve complete meconium evacuation and full enteral feeding will be analyzed using Kaplan–Meier curves and log-rank tests. In the Kaplan–Meier analysis, the computation of the median time holds significant methodological importance.

Precisely, the median time to accomplish complete meconium evacuation and full enteral feeding are ascertained as the time point at which 50% of the study participants reach these predefined endpoints.

14.3 Analysis of Secondary Outcomes

The secondary outcomes will include the hospitalisation days, weight at the time of discharge, and duration of TPN. The outcomes will be analyzed using an independent sample t-test or Mann–Whitney test. Meanwhile, a multiple linear regression model will be used to analyze the factors affecting primary outcomes, including gender, birth weight, history of a mother's disease, etc. Outcomes are presented as median (IQR). For outcome differences between the treatment and control arms, the median difference and 95% CI will be assessed using the Mann–Whitney U test and the Hodges–Lehmann method.

14.4 Analysis of safety

Safety will be assessed and the incidence of safety events will be analyzed using descriptive statistics. Safety events include ROP of any stage, (IVH, NEC, BPD, LOS, and colorectal and anal injuries, and death.

14.5 Subgroup Analysis

For all aforementioned statistical analyses, stratified subgroup analyses will be conducted based on gestational age to compare specific intra-group outcomes. Gestational age categories are 23 weeks ≤ gestational age < 28 weeks, 28 weeks ≤ gestational age < 29 weeks and 29weeks ≤ gestational age < 30 weeks This approach is employed to assess nuanced differences within distinct gestational age subgroups, enabling a comprehensive evaluation of outcomes tailored to the developmental stage of the fetus.

14.6 Statistical software

A two-sided P < 0.05 will be considered statistically significant. All analysis will be performed in SAS version 9.2 (SAS Institute Inc., Cary, NC, USA) and SPSS version 22.0 (IBM Inc., Chicago, IL, USA) software. Interim analyses are not planned.

15. Discussion

Due to the large number of premature infants being born worldwide, countries are facing an enormous medical burden. Feeding is a significant challenge for premature infants and establishing full enteral feeding is an important goal in their care. Earlier full enteral feeding is associated with fewer failures of postpartum growth. Unfortunately, any delay in meconium excretion is related to a delay in transition to full enteral feeding. Compared with term infants, many premature infants pass the first meconium only after considerable delay. Times for the evacuation of the first and last meconium is critical for gastrointestinal function and feeding tolerance. If meconium evacuation can be expedited through the use of an effective enema, this may lead to a faster transition to full enteral feeding and a decreased reliance on intravenous nutrition. However, the available evidence is inconclusive. In contrast to other enemas, the amount of magnesium salts in breast milk is high and relatively stable, which can promote gastrointestinal peristalsis and help meconium evacuation[21,22]. In addition, breast milk can reduce the risk of enterovirus infection and balance the intestinal flora of premature infants[23,24]. Therefore, the trial will collect all participants' data to determine whether this trial has potential clinical significance and can provide reliable evidence for clinical intervention. The results may yield a new, safe, inexpensive, and easy-to-use intervention to effectively evacuate meconium in preterm infants.

To the best of our knowledge, the major strength of this randomized controlled trial is to evaluate, for the first time, the effectiveness of a breast milk enema in promoting complete meconium evacuation and reach full enteral feeding among preterm and extremely preterm infants. Consequently, the results will provide critical information regarding a new enema to effectively evacuate meconium in preterm infants.

The study protocol has several limitations. First, some infants achieving complete meconium evacuation may withdraw before observing the time to reach full enteral feeding. We will further analyze and design a more comprehensive investigation

based on this study. Second, this trial is a single-center clinical trial. Therefore, the representativeness of the participants is limited to some extent. Finally, some mothers may be unable to produce enough breast milk in early postpartum, which may slow down trial progress and prolong the time to recruit the estimated sample size.

References

[1] Blencowe H, Cousens S, Oestergaard MZ, *et al*. National, regional, and worldwide estimates of preterm birth rates in the year 2010 with time trends since 1990 for selected countries: a systematic analysis and implications. Lancet 2012; 379:2162-2172.

[2] Goldenberg RL, Culhane JF, Iams JD, *et al*. Epidemiology and causes of preterm birth. Lancet 2008; 371:75-84.

[3] Chathas MK, Paton JB, Fisher DE. Percutaneous central venous catheterization. Three years' experience in a neonatal intensive care unit. Am J Dis Child 1990; 144:1246-1250.

[4] Unger A, Goetzman BW, Chan C, et al. Nutritional practices and outcome of extremely premature infants. Am J Dis Child 1986; 140:1027-1033.

[5] Stoll BJ, Gordon T, Korones SB, et al. Late-onset sepsis in very low birth weight neonates: a report from the National Institute of Child Health and Human Development Neonatal Research Network.J Pediatr1996; 129:63-71.

[6] Dimmitt RA, Moss RL. Meconium diseases in infants with very low birth weight. Semin Pediatr Surg 2000; 9:79-83.

[7] Krasna IH, Rosenfeld D, Salerno P. Is it necrotizing enterocolitis, microcolon of prematurity, or delayed meconium plug? A dilemma in the tiny premature infant. J Pediatr Surg 1996; 31:855-858.

[8] Bekkali N, Hamers SL, Schipperus MR, et al. Duration of meconium passage in preterm and term infants. Arch Dis Child Fetal Neonatal Ed 2008; 93:F376-379.

[9] Mihatsch WA, Franz AR, Lindner W, et al. Meconium passage in extremely low birthweight infants and its relation to very early enteral nutrition. Acta Paediatr 2001; 90:409-411.

[10] Haiden N, Norooz F, Klebermass Schrehof K, et al. The effect of an osmotic contrast agent on complete meconium evacuation in preterm infants. Pediatrics 2012; 130:e1600-1606.

[11] de Pipaón Marcos MS, Montes Bueno MT, SanJosé B, et al. Acquisition of full enteral feeds may depend on stooling pattern in very premature infants. J Perinat Med 2012; 40:427-431.
[12] Shim S, Kim H, Kim D, et al. Induction of early meconium evacuation promotes feeding tolerance in very low birth weight infants. Neonatology 2007; 92:67-72.
[13] Fakhri M, Farhadi R, Mousavinasab N, et al. Preventive effect of purgative manna on neonatal jaundice: A double blind randomized controlled clinical trial. J Ethnopharmacol 2019; 236:240-249.
[14] Shim SY, Kim HS, Kim DH, et al. Induction of early meconium evacuation promotes feeding tolerance in very low birth weight infants. Neonatology 2007; 92:67-72.
[15] Solaz-García AJ, Segovia-Navarro L, Rodríguez de Dios-Benlloch JL, et al. Prevention of meconium obstruction in very low birth weight preterm infants. Enferm Intensiva 2019; 30:72-77.
[16] Kamphorst K, Sietsma Y, Brouwer AJ, et al. Enemas, suppositories and rectal stimulation are not effective in accelerating enteral feeding or meconium evacuation in low birth weight infants: A systematic review. Acta Paediatrica 2016; 105:1280-1287.
[17] Michikata K, Kodama Y, Kaneko M, et al. Oral diatrizoate acid for meconium-related ileus in extremely preterm infants. Pediatr Int 2018; 60:714-718.
[18] Livingston MH, Shawyer AC, Rosenbaum PL, et al. Glycerin enemas and suppositories in premature infants: a meta-analysis. Pediatrics 2015; 135:1093-1106.
[19] Deshmukh M, Balasubramanian H, Patole S. Meconium evacuation for facilitating feed tolerance in preterm neonates: A Systematic Review and Meta-Analysis. Neonatology 2016; 110:55-65.
[20] Du ZM, Meng T, Lin Z. Comparison between high osmotic pressure breast milk and isosmotic pressure breast milk fed infants with urine mAlb and RBP. Journal of practical medical techniques 2006; (02):211-212. In Chinese.

[21] Bell MJ, Ternberg JL, Feigin RD, et al. Neonatal necrotizing enterocolitis. Therapeutic decisions based upon clinical staging. Ann Surg 1978; 187:1–7.

[22] Anabrees J, Shah VS, AlOsaimi A, et al. Glycerin laxatives for prevention or treatment of feeding intolerance in very low birth weight infants.Cochrane Database Syst Rev 2015; 9:CD010464.

[23] International Committee for the Classification of Retinopathy of Prematurity. The International Classification of Retinopathy of Prematurity revisited. Arch Ophthalmol 2005; 123:991–999.

[24] Papile LA, Burstein J, Burstein R, et al. Incidence and evolution of subependymal and intraventricular hemorrhage: a study of infants with birth weights less than 1,500 gm. J Pediatr 1978; 92:529–534.
