# Supplementary material for: Breast Milk Enema and Meconium Evacuation Among Preterm Infants: A Randomized Clinical Trial
Source: JAMA Netw Open. 2024 Apr 22;7(4):e247145. doi: 10.1001/jamanetworkopen.2024.7145 (PMC11981638; doi:10.1001/jamanetworkopen.2024.7145)
Supplement: Supplement 2. — eTable 1. Safety Outcomes Among Overall Participants and in Subgroup Analyses for the ITT Set eTable 2. Sensitivity Analyses on Primary Outcomes eTable 3. Adverse Events Leading to Discontinuation Among All Participants eTable 4. Baseline Characteristics of Infants and Their Mothers eTable 5. Effect of Breast Milk Enema on Primary and Secondary Outcomes Among Overall Participants and in Subgroup Analyses eTable 6. Safety Outcomes Among Overall Participants and in Subgroup Analyses for the PP Set [file jamanetwopen-e247145-s002.pdf]

Supplementary Online Content

Zheng L, Gai L, Wu Y, et al. Breast milk enema and meconium evacuation among preterm infants: a randomized clinical trial. *JAMA Netw Open.* 2024;7(4):e247145. doi:10.1001/jamanetworkopen.2024.7145

eTable 1. Safety Outcomes Among Overall Participants and in Subgroup Analyses for the ITT Set
eTable 2. Sensitivity Analyses on Primary Outcomes
eTable 3. Adverse Events Leading to Discontinuation Among All Participants
eTable 4. Baseline Characteristics of Infants and Their Mothers
eTable 5. Effect of Breast Milk Enema on Primary and Secondary Outcomes Among Overall Participants and in Subgroup Analyses
eTable 6. Safety Outcomes Among Overall Participants and in Subgroup Analyses for the PP Set

This supplementary material has been provided by the authors to give readers additional information about their work.

© 2024 Zheng L et al. *JAMA Network Open.*

eTable 1. Safety Outcomes Among Overall Participants and in Subgroup Analyses for the ITT Set

	Total (n=286)			23-28 weeks (n=78)			28 -29 weeks (n=100)			29 -30weeks (n=108)		
	NS (n=145)	BM (n=141)	Risk Ratio (95%CI)	NS (n=39)	BM (n=39)	Risk Ratio (95%CI)	NS (n=52)	BM (n=48)	Risk Ratio (95%CI)	NS (n=54)	BM (n=54)	Risk Ratio (95%CI)
Discontinuation due to AE, n (%)	2 (1.4)	11 (7.8)	5.66 (1.28-25.06)	2 (5.1)	4 (10.3)	2.00 (0.39-10.29)	0 (0)	1 (2.1)	-	0 (0)	6 (11.1)	-
BPD, n (%)	113 (77.9)	107 (75.9)	1.03 (0.90-1.17)	35 (89.7)	33 (84.6)	1.06 (0.89-1.26)	45 (86.5)	39 (81.3)	1.07 (0.90-1.27)	33 (61.1)	35 (64.8)	0.94(0.71-1.26)
LOS, n (%)	49 (33.8)	43 (30.5)	1.11 (0.79-1.55)	11 (28.2)	9 (23.1)	1.22 (0.57-2.62)	23 (44.2)	20 (41.7)	1.06 (0.68-1.67)	15 (27.8)	14 (25.9)	1.07(0.58-2.00)
ROP , n (%)	41 (28.3)	31 (22.0)	1.29 (0.86-1.93)	25 (64.1)	17 (43.6)	1.47 (0.96-2.26)	12 (23.1)	11 (22.9)	1.01 (0.49-2.07)	4 (7.4)	3 (5.6)	1.33(0.31-5.68)
IVH, n (%)	10 (6.9)	5 (3.5)	1.95 (0.68-5.55)	3 (7.7)	1 (2.6)	3.00 (0.33-27.60)	3 (5.8)	1 (2.1)	2.77 (0.30-25.73)	4 (7.4)	3 (5.6)	1.33(0.31-5.68
NEC, n (%)	11 (7.6)	9 (6.4)	1.19 (0.51-2.78)	1 (2.6)	2 (5.1)	0.50 (0.05-5.30)	6 (11.5)	4 (8.3)	1.39 (0.42-4.61)	4 (7.4)	3 (5.6)	1.33(0.31-5.68)
Bloody stools, n (%)	1 (0.7)	3 (2.1)	0.32 (0.03-3.08)	1 (2.6)	1 (2.6)	1.00 (0.07-15.43)	0 (0.0)	0 (0.0)	-	0 (0.0)	2 (3.7)	-
Colorectal and anal injuries, n (%)	0 (0.0)	4 (2.8)	-	0 (0.0)	0 (0.0)	-	0 (0.0)	0 (0.0)	-	0 (0.0)	4 (7.4)	-
Mortality, n (%)	1 (0.7)	1 (0.7)	0.97 (0.05-15.40)	0 (0.0)	1 (2.6)	-	0 (0.0)	0 (0.0)	-	1 (1.9)	0 (0.0)	-

Abbreviations：NS, Normal Saline; BM, breast milk; BPD, bronchopulmonary dysplasia; LOS, late onset sepsis; ROP, retinopathy of prematurity; IVH, intraventricular hemorrhage; NEC, necrotizing enterocolitis.

© 2024 Zheng L et al. *JAMA Network Open.*

eTable 2. Sensitivity Analyses on Primary Outcomes

	Total				23-28 weeks				28 -29 weeks				29 -30weeks			
	NS	BM	Estimated difference (95%CI)	p-value	NS	BM	Estimated difference (95%CI)	p-value	NS	BM	Estimated difference (95%CI)	p-value	NS	BM	Estimated difference (95%CI)	p-value
**Sensitivity Analysis 1[a]**																
Time to achieve complete meconium evacuation, days	13.7 (10.7-17.5)	11.7 (9.7-15.1)	-1.8 (-2.8~-0.7)	**0.004**	12.8 (9.5-16.0)	11.7 (9.7-14.2)	-1.2 (-3.3~0.9)	0.50	14.8 (11.5-17.9)	12.5 (9.8-15.3)	-2.3 (-4.0~-0.6)	**0.01**	13.7 (10.3-17.8）	11.4 (8.8-15.1)	-1.5 (-3.3~0.4)	0.23
Time to achieve full enteral feeding, days	35.5 (25.7-47.0)	31.2 (22.0-46.9)	-3.0 (-6.5~0.7)	0.23	46.5 (32.9-66.9)	36.8 (25.5-55.1）	-16.0 (-7.2~2.9)	0.27	35.5 (27.5-44.8)	31.8 (25.8-43.8)	-2.2 (-8.0~3.3)	0.90	26.8 (21.8-36.7)	24.2 (19.7-45.8)	-2.3 (-6.1~2.7)	0.72
**Sensitivity Analysis 2**																
Time to achieve complete meconium evacuation, days	13.6 (10.5-13.6)	11.0 (8.9-14.0)	-2.5 (-3.5~-1.4)	**<0.001**	12.5 (9.0-15.8)	10.6 (8.8-12.7)	-1.6 (-3.5~0.3)	0.19	14.8 (11.5-17.9)	12.4 (9.7-15.2)	-2.5 (-4.1~-0.9)	**0.004**	13.7 (10.3-17.8）	10.8 (7.6-13.7)	-2.9 (-4.6~-1.1)	**0.002**
Time to achieve full enteral feeding, days	35.4 (25.6-46.6)	28.7 (20.7-41.5)	-6.2 (-10.1~-2.9)	**0.002**	45.8 (31.9-64.1)	30.6 (23.8-46.9）	-12.0 (-20.9~-3.5)	**0.01**	35.5 (27.5-44.8)	31.6 (24.9-42.3)	-3.1 (-9.4~2.3)	0.53	26.8 (21.8-36.7)	21.8 (16.3-32.4)	-6.0 (-10.9~-2.0)	**0.01**

Data are presented as median(Q1-Q3). The median difference and 95%CI were assessed using the Mann–Whitney U test and the Hodges–Lehmann method. NS = Normal Saline; BM = breast milk; TPN = total parenteral nutrition.

[a] For the infants who discontinued due to AE, sensitivity analysis 1 used worst outcome imputation, and sensitivity analysis 2 used the last observation time.

© 2024 Zheng L et al. *JAMA Network Open.*

eTable 3. Adverse Events Leading to Discontinuation Among All Participants

	Gestational age	Group	AE leading to discontinuation [a]
1	23-28w	NS	Bloody stools
2	23-28w	NS	NEC
3	23-28w	BM	Congenital Heart Disease
4	23-28w	BM	Bloody stools
5	23-28w	BM	NEC
6	23-28w	BM	NEC
7	28-29w	BM	NEC
8	29-30w	BM	Colorectal and anal injuries
9	29-30w	BM	Colorectal and anal injuries
10	29-30w	BM	Colorectal and anal injuries
11	29-30w	BM	Bloody stools
12	29-30w	BM	Bloody stools
13	29-30w	BM	Surgery

Abbreviations: AE, Adverse Events; Normal Saline; BM, breast milk; NEC, necrotizing enterocolitis.
[a] These AEs leading to the discontinuations(NEC, colorectal and anal injuries, and bloody stools) were also included in the counting and statistical analysis in eTable 1.

© 2024 Zheng L et al. *JAMA Network Open.*

eTable 4. Baseline Characteristics of Infants and Their Mothers

	Total (n=252)		23-28 weeks (n=65)		28 -29 weeks (n=92)		29 -30weeks (n=95)	
	NS (n=133)	BM (n=119)	NS (n=35)	BM (n=30)	NS (n=48)	BM (n=44)	NS (n=50)	BM (n=45)
Mother's age, y, mean±SD	32.1±4.4	31.9±5.1	32.5±4.3	31.0±5.2	31.5±4.1	31.4±4.1	32.2±4.9	33.1±5.7
Education, n (%)								
Less than a high school diploma	35 (26.3)	23 (19.3)	8 (22.9)	6 (20.0)	15 (31.3)	6 (13. 6)	12 (24.0)	11 (24.4)
High school degree	25 (18.8)	29 (24.4)	8 (22.9)	5 (16.7)	9 (18.8)	13 (29.5)	8 (16.0)	11 (24.4)
Associate degree	29 (21.8)	23 (19.3)	6 (17.1)	5 (16.7)	11 (22.9)	9 (20.5)	12 (24.0)	9 (20.0)
Bachelor's degree and above	44 (33.1)	44 (37.0)	13 (37.1)	14 (46.7)	13 (27.1)	16 (36.4)	18 (36.0)	14 (31.1)
Cesarean, n (%)	82 (61.7)	75 (63.0)	21 (61.8)	13 (43.3)	28 (58.3)	30 (68.2)	33 (66.0)	32 (71.1)
Number of pregnancies, median (Q1-Q3)	2.0 (1.0-3.0)	2.0 (1.0-3.0)	2.0 (2.0)	2.0 (1.0-3.0)	2.0 (1.0-2.0)	2.0 (1.0-3.0)	2.0 (1.0-3.0)	2.0 (1.0-3.0)
Parity, median (Q1-Q3)	1.0 (1.0-2.0)	1.0 (1.0-2.0)	1.0 (1.0)	1.0 (1.0-2.0)	1.0 (1.0-2.0)	1.0 (1.0-2.0)	1.0 (1.0-2.0)	2.0 (1.0-2.0)
Pregnancy Induced Hypertension, n (%)	34 (25.6)	31(26.1)	7 (20.0)	6 (20.0)	12 (25.0)	17 (38.6)	15 (30.0)	8 (17.8)
Diabetes mellitus, n (%)	24 (18.0)	18(15.1)	5 (14.3)	1 (3.3)	10 (20.8)	8 (18.2)	9 (18.0)	9 (20.0)
Medication use during pregnancy, n (%)	59 (44.4)	64(54.8)	16 (45.7)	15 (50.0)	21 (43.8)	29 (65.9)	22 (44.0)	20 (44.4)
Male, n (%)	72(54.1)	73(61.3)	23 (65.7)	19 (63.3)	22 (45.8)	29 (65.9)	23 (46.0)	20 (44.4)
Gestational age, days, mean±SD	199.0±7.1	199.2±7.7	189.4±4.9	188.4±5.3	198.8±1.7	199.3±2.1	205.8±2.2	206.3±2.1
Birth weight, g, mean±SD	1123.7±213.6	1136.9±253.6	974.3±145.8	940.4±175.2	1104.2±211.0	1116.6±248.4	1247.1±183.4	1287.9±204.8
Apgar score, median (Q1-Q3)								
1 min	7.0 (6.0-8.0)	7.0 (6.0-8.0)	6.0 (3.0)	6.0 (5.0-7.3)	7.0 (6.0-8.0)	7.0 (6.0-8.0)	8.0 (6.0-9.0)	7.0 (6.0-8.0)
5 min	9.0 (8.0-9.0)	9.0 (8.0-9.0)	8.0 (2.0)	8.0 (8.0-9.0)	9.0 (8.0-9.0)	9.0 (8.0-9.0)	9.0 (8.0-9.0)	9.0 (8.5-9.0)

Data are n (%) or mean (SD) or median(Q1-Q3) and include all patients in the full analysis set, unless indicated otherwise.

© 2024 Zheng L et al. *JAMA Network Open.*

eTable 5. Effect of Breast Milk Enema on Primary and Secondary Outcomes Among Overall Participants and in Subgroup Analyses

	Total				23-28 weeks				28 -29 weeks				29 -30weeks			
	NS	BM	Estimated difference (95%CI)	p-value	NS	BM	Estimated difference (95%CI)	p-value	NS	BM	Estimated difference (95%CI)	p-value	NS	BM	Estimated difference (95%CI)	p-value
Time to achieve complete meconium evacuation, days	13.7 (10.6~16.9)	11.6 (9.5~14.1)	-2.1 (-3.1~-1.0)	**<0.001**	12.8 (9.5~15.8)	10.9 (9.3~1.9)	-1.4 (-3.3~0.6)	0.31	14.8 (11.5~17.9)	12.6 (9.9~15.3)	-2.1 (-3.8~-0.5)	**0.02**	13.7 (10.3~17.8）	11.0 (8.8~13.7)	-2.7 (-4.3~-0.8)	**0.008**
Time to achieve full enteral feeding, days	35.4 (25.6~46.8)	29.2 (21.5~42.0)	-4.7 (-8.2~-1.3)	**0.007**	46.5 (31.9~65.0)	31.2 (24.6~47.3）	-11.0 (-20.1~-2.2)	**0.02**	35.5 (27.5~44.8)	31.4 (24.9~43.3)	-2.6 (-8.3~2.9)	0.69	26.8 (21.8~36.7)	22.7 (18.7~32.8)	-4.1 (-8.3~0.1)	0.10
Duration of TPN, days	35.8 (25.7~50.1)	30.2 (21.7~42.6)	-4.7 (-8.4~-1.1)	**0.01**	47.5 (34.0~65.0)	31.3 (24.0~48.9)	-11.5 (-20.5~-2.9)	**0.006**	35.5 (27.4~45.0)	33.6 (24.9~43.5)	-1.5 (-7.5~4.1)	0.60	27.6 (21.7~39.6)	22.9 (18.8~36.0)	-4.3 (-9.0~0.2)	0.06
Hospitalization days for infant,days	62.0 (48.0~77.0)	60.0 (48.0~74.0)	-1.0 (-6.0~5.0)	0.79	63.0 (48.0~79.0)	69.5 (48.5~83.0)	3.0 (-8.0~15.0)	0.60	61.0 (46.0~78.0)	64.5 (53.8~74.3)	6.0 (-3.0~15.0)	0.22	63.0 (48.8~75.0)	51.0 (43.0~66.0)	-8.5 (-18.0~-1.0)	0.04
Weight at discharge, g	2240.0 (1990.0~2557.5)	2270.0 (2020.0~2525.0)	20.0 (-70.0~100.0)	0.68	2550.0 (2220.0~2850.0)	2410.0 (2085.0~2855.0)	-70.0 (-300.0~150.0)	0.50	2140.0 (1940.0~2440.0)	2245.0 (2015.0~2557.5)	60.0 (-100.0~210.0)	0.45	2165.0 (1957.5~2365.0)	2230.0 (2015.0~2400.0)	50.0 (-70.0~160.0)	0.36

Abbreviations：NS, Normal Saline; BM, breast milk; TPN, total parenteral nutrition.

© 2024 Zheng L et al. *JAMA Network Open.*

eTable 6. Safety Outcomes Among Overall Participants and in Subgroup Analyses for the PP Set

	Total (n=252)			23-28 weeks (n=65)			28 -29 weeks (n=92)			29 -30weeks (n=95)		
	NS (n=133)	BM (n=119)	Risk Ratio (95%CI)	NS (n=35)	BM (n=30)	Risk Ratio (95%CI)	NS (n=48)	BM (n=44)	Risk Ratio (95%CI)	NS (n=50)	BM (n=45)	Risk Ratio (95%CI)
BPD, n (%)	111 (83.5)	103 (86.6)	0,96 (0.87-1.07)	35 (100.0)	30 (100.0)	-	43 (52.4)	39 (47.6)	1.01 (0.88-1.17)	33 (66.0)	34 (75.6)	0.87 (0.67-1.13)
LOS, n (%)	48 (36.1)	39 (32.8)	1.10 (0.78-1.55)	11 (31.4)	8 (26.7)	1.18 (0.55-2.54)	22 (45.8)	19 (43.2)	1.06 (0.67-1.68)	15 (30.0)	12 (26.7)	1.13 (0.59-2.14)
ROP, n (%)	41 (30.8)	31 (26.1)	1.18 (0.80-1.76)	25 (71.4)	17 (56.7)	1.26 (0.87-1.84)	12 (25.0)	11 (25.0)	1.00 (0.49-2.03)	4 (8.0)	3 (6.7)	1.20 (0.28-5.07)
IVH, n (%)	9 (6.8)	4 (3.4)	2.01 (0.64-6.37)	2 (5.7)	1 (3.3)	1.71 (0.16-17.98)	3 (6.3)	1 (2.3)	2.75 (0.30-25.47)	4 (8.0)	2 (4.4)	1.80 (0.35-9.36)
NEC, n (%)	10 (7.5)	6 (5.0)	1.49 (0.56-3.98)	0 (0.0)	0 (0.0)	-	6 (12.5)	3 (6.8)	1.83 (0.49-6.89)	4 (8.0)	3 (6.7)	1.20 (0.28-5.07)
Bloody stools, n (%)	0 (0.0)	1 (0.8)	-	0 (0.0)	1 (3.3)	-	0 (0.0)	0 (0.0)	-	0 (0.0)	0 (0.0)	-
Colorectal and anal injuries, n (%)	0 (0.0)	1 (0.8)	-	0 (0.0)	0 (0.0)	-	0 (0.0)	0 (0.0)	-	0 (0.0)	1 (2.2)	-

Abbreviations：NS, Normal Saline; BM, breast milk; BPD, bronchopulmonary dysplasia; LOS, late onset sepsis; ROP, retinopathy of prematurity; IVH, intraventricular hemorrhage; NEC, necrotizing enterocolitis.

© 2024 Zheng L et al. *JAMA Network Open.*
