# Supplementary material for: Breast Milk Enema and Meconium Evacuation Among Preterm Infants: A Randomized Clinical Trial
Source: JAMA Netw Open. 2024 Apr 22;7(4):e247145. doi: 10.1001/jamanetworkopen.2024.7145 (PMC11981638; doi:10.1001/jamanetworkopen.2024.7145)
Supplement: Supplement 3. — Data Sharing Statement [file jamanetwopen-e247145-s003.pdf]

Data Sharing Statement

Zheng. Breast Milk Enema and Meconium Evacuation Among Preterm Infants. *JAMA Netw Open*. Published April 22, 2024. doi:10.1001/jamanetworkopen.2024.7145

Data
Data available: Yes
Data types: Deidentified participant data, Data dictionary
How to access data: liqiangzheng@126.com
When available: With publication

Supporting Documents
Document types: None

Additional Information
Who can access the data: researchers whose proposed use of the data has been approved
Types of analyses: for a specified purpose
Mechanisms of data availability: with a signed data access agreement
